# Using gridded population and quadtree sampling units to support survey sample design in low-income settings

**DOI:** 10.1186/s12942-020-00205-5

**Published:** 2020-03-26

**Authors:** Sarchil Hama Qader, Veronique Lefebvre, Andrew J. Tatem, Utz Pape, Warren Jochem, Kristen Himelein, Amy Ninneman, Philip Wolburg, Gonzalo Nunez-Chaim, Linus Bengtsson, Tomas Bird

**Affiliations:** 1 WorldPop, Geography and Environmental Science, University of Southampton, University Road, Southampton, UK; 2 Natural Resources Department, College of Agricultural Engineering Sciences, University of Sulaimani, Sulaimani, Kurdistan Region Iraq; 3 Flowminder Foundation, Roslagsgatan 17, Stockholm, Sweden; 4 World Bank, Washington DC, USA

## Abstract

**Background:**

Household surveys are the main source of demographic, health and socio-economic data in low- and middle-income countries (LMICs). To conduct such a survey, census population information mapped into enumeration areas (EAs) typically serves a sampling frame from which to generate a random sample. However, the use of census information to generate this sample frame can be problematic as in many LMIC contexts, such data are often outdated or incomplete, potentially introducing coverage issues into the sample frame. Increasingly, where census data are outdated or unavailable, modelled population datasets in the gridded form are being used to create household survey sampling frames.

**Methods:**

Previously this process was done by either sampling from a set of the uniform grid cells (UGC) which are then manually subdivided to achieve the desired population size, or by sampling very small grid cells then aggregating cells into larger units to achieve a minimum population per survey cluster. The former approach is time and resource-intensive as well as results in substantial heterogeneity in the output sampling units, while the latter can complicate the calculation of unbiased sampling weights. Using the context of Somalia, which has not had a full census since 1987, we implemented a quadtree algorithm for the first time to create a population sampling frame. The approach uses gridded population estimates and it is based on the idea of a quadtree decomposition in which an area successively subdivided into four equal size quadrants, until the content of each quadrant is homogenous.

**Results:**

The quadtree approach used here produced much more homogeneous sampling units than the UGC (1 × 1 km and 3 × 3 km) approach. At the national and pre-war regional scale, the standard deviation and coefficient of variation, as indications of homogeneity, were calculated for the output sampling units using quadtree and UGC 1 × 1 km and 3 × 3 km approaches to create the sampling frame and the results showed outstanding performance for quadtree approach.

**Conclusion:**

Our approach reduces the manual burden of manually subdividing UGC into highly populated areas, while allowing for correct calculation of sampling weights. The algorithm produces a relatively homogenous population counts within the sampling units, reducing the variation in the weights and improving the precision of the resulting estimates. Furthermore, a protocol of creating approximately equal-sized blocks and using tablets for randomized selection of a household in each block mitigated potential selection bias by enumerators. The approach shows labour, time and cost-saving and points to the potential use in wider contexts.

## Background

In all countries, surveys and census data are the main source of demographic, health and socio-economic data. In particular, household surveys are the main means of providing detailed health/socio-economic information in a timely fashion, since the burden of conducting of the full census is labor and cost-prohibitive. In low- and middle-income countries (LMICs), surveys such as the Living Standard Measurement Surveys (LSMS), the UN’s Multiple Indicator Cluster Survey (MICS) and the Demographic and Health Surveys (DHS) have been routinely implemented since the 1980s [1–3]. Household surveys typically rely on census data as a sampling frame [4]. The use of census data as a source of the sampling frame is critical as it allows survey designers to efficiently allocate their sample across areas or populations as well as identify groups typically under-represented or rare [5]. The sampling frame also represents the best-known distribution of the population at the time of the sample selection, making it a critical input to the weight calculations. However, the use of census data as the sampling frame is problematic in many countries around the world, particularly in LMICs, since their census data are often outdated, incomplete, or missing, or inaccessible. For example, Afghanistan has not conducted a full national census since 1979 [6], and Somalia since 1987 [7].

Where full census data are not available in a country, gridded population datasets have emerged over the last decade as a potential alternative to building household survey sampling frames [8–11]. Gridded population data are usually produced by models to give estimate counts of population density in uniform grid cells. Currently available gridded population datasets are derived with models that either disaggregate census data or predict population density based on a subset of the population, and can incorporate information from spatial covariates, such as land cover type, road infrastructure, nightlight intensity and settlement areas [12]. Several gridded population datasets with different spatial resolutions are available in LMICs including WorldPop [13], Gridded Population of the World (GPWv4) [14], Global Human Settlement Population Grid (GHS-POP) [15], High-Resolution Settlement Layer (HRSL) [16], Global Rural–Urban Mapping Project (GRUMP) [17], LandScan [18], and Demobase Population datasets [19].

From a survey practitioner perspective, a key difference between gridded population data and census data is that grid cells are uniform in size but variable in population totals whereas census EAs vary in size but have similar population totals. From a sample design efficiency perspective, similar population estimates in the Primary Sampling Unit (PSU)—which are the grid cell and EA, respectively—equally sized population leads to greater precision in the resulting estimates. Because of this property, gridded population estimates can be useful for survey sample design even when a census frame exists because it can be used to update older census datasets, subdividing EAs that have grown too large. Similarly, being able to identify areas which have seen a net decrease in population can also increase the efficiency over an outdated census.

In practice, only two gridded population datasets have been used for household survey sampling: LandScan [20] and WorldPop [12]. For instance, Galway et al. [10] used LandScan to generate 1 × sampling units with probability proportional to size (PPS) and selected one household in one building and performed a random walk [10]. A grid-based sample design framework for household surveys using the WorldPop dataset was conducted in DRC and a nonparametric estimator was applied to evaluate the sample design and determine sample size estimation [21]. In the context of creating household sampling frames, WorldPop may offer three advantages to LandScan. First, WorldPop is available at a fine geographic scale that suits the scale at which household survey activities occur; in densely settled areas, each 100 × 100 m WorldPop cell contains a maximum of hundreds, rather than thousands of households found in some 1 × 1 km LandScan cells. In surveys where LandScan was used as a sampling frame, intensive manual segmentation [22] or complex automated segmentation [23] was required. Second, WorldPop is a model of the residential population while LandScan is a model of the 24-h ambient population—the average of the day-time commuter population and night-time residential population [13]. Third, WorldPop routinely publishes accuracy assessments [13, 24–27].

Considering the lack of ground truth data in Somalia, it was a challenge to assess the accuracy of the existing available gridded population datasets at the time of this study. In addition, such analysis requires full research commitment, and this might not fall within the scope of this paper. However, gridded population datasets are being produced for countries with similar conditions [28–30]. Overall the accuracy and reliability of these approaches are increasing as new research into modelling approaches and predictive covariates. For instance, in collaboration with other partners, WorldPop has produced a disaggregated population dataset to 100m × 100m grid square in Afghanistan and accuracy measuring observed vs predicted population numbers showed correlation coefficients of > 0.95 at the district and province level [28]. Recently, under the leadership of WorldPop within the Geo-Referenced Infrastructure and Demographic Data for Development (GRID^3^) project, high resolution population estimates were produced for DRC and Nigeria and uncertainty in the population estimates within each 100 m grid cell was calculated across the predicted population areas [29, 30]. While we acknowledge that modelled population estimates will never be as good as actual census counts, the results are increasingly being used in contexts where such census data are simply unavailable.

In previous gridded population surveys, practitioners either started with UGCs (e.g. 1 km × 1 km) and segmented, sometimes performing a second stage of sampling of smaller units within the cell [9, 22, 23], an extremely time and/or resource-intensive process. Other surveys have sampled small grid cells (e.g. 100 m × 100 m) and included neighbouring areas after initial cells were selected to ensure a minimum population per sampling unit [11]. This approach requires the calculation of a complex adaptive sample probability weight or risks incorrect weights and estimates [31]. In terms of available methods, there have been limited efforts made to develop tools and approaches for creating a population sampling frame from gridded population estimates (see GridSample [32] and Geo-sampling tool [33] for notable exceptions). Our approach has improved and solved some of the challenges that have faced these efforts. Firstly, it allows an accurate calculation of sampling probability weights compared to GridSample. Secondly, in our approach, we can define the population and area constraints based on our requirements, resulting in a user-friendly sampling frame with much-improved population homogeneity within the sampling units. Therefore, an ideal gridded population sampling frame would efficiently group cells into areas of varying size with similar population totals before sampling.

Here, we sought to develop a sampling frame for a household sampling approach in Somalia where the stationary population is overwhelmingly rural, and upwards of a third of the population is nomadic [7]. In addition, in large parts of the country, it is not possible to conduct a complete household listing due to security considerations for field staff [34, 35]. This paper describes a novel approach to pre-define a survey population sampling frame using gridded population data. The goal was to support the design and implementation of a household socio-economic survey that was representative of urban, rural, the internally displaced population (IDP) and nomadic people in Somalia. Our approach uses GIS techniques, several datasets, and a quadtree algorithm [36] to recursively divide the country into areas with homogenous population sizes. We provide example outputs, output evaluation criteria, and clear steps in an appendix to replicate these methods.

## Methodology

### General approach

**Fig. 1:**
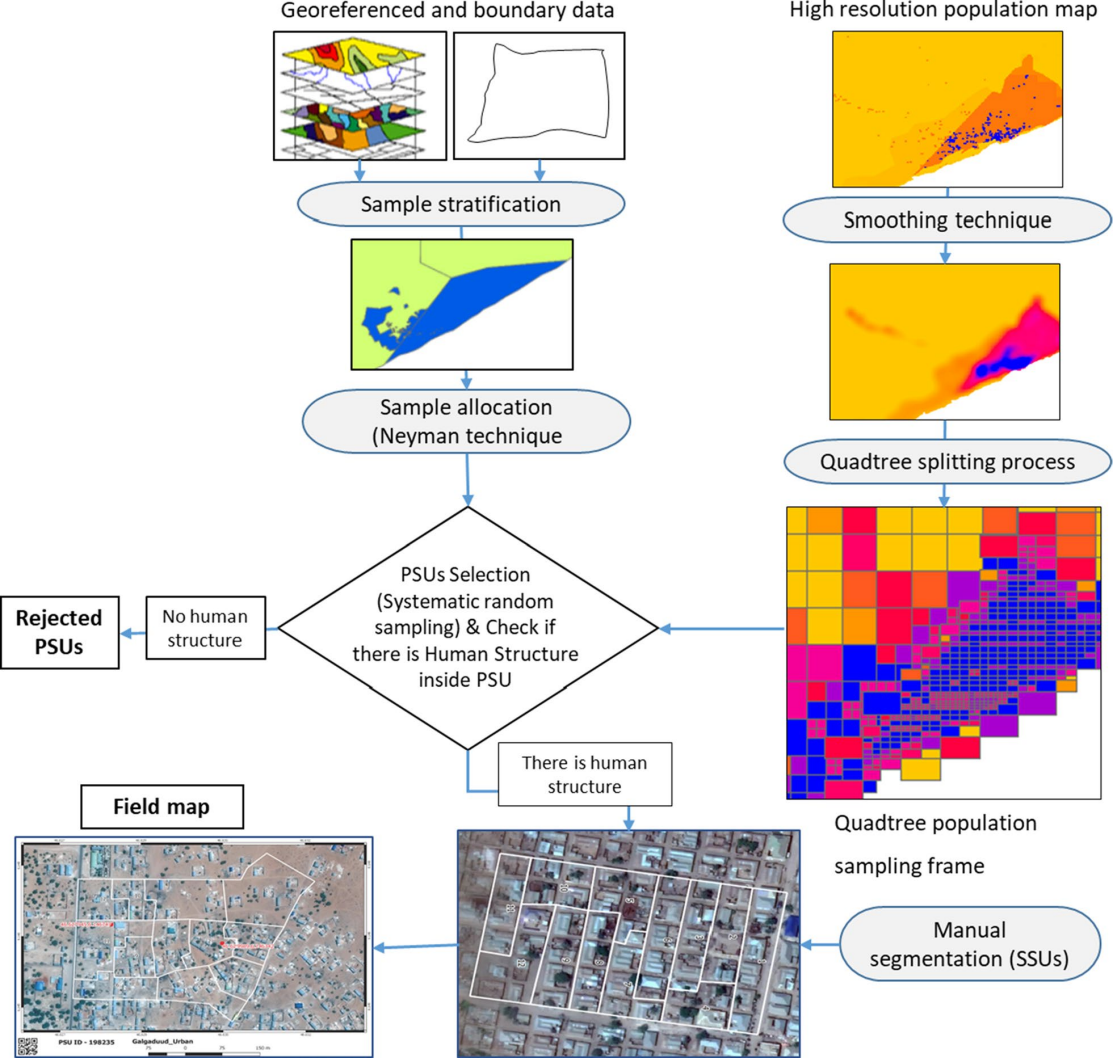
Schematic diagram showing the methodology adopted in this research

Here we describe the process for deriving enumeration areas with desirable area and population characteristics based on gridded population data. We used high-resolution (100 m) estimates of population density for Somalia in 2015 [12] as the basis for our process. Because we needed to stratify the population into distinct types (based on administrative boundaries and population types). We also obtained vector data of administrative boundaries, IDP camp locations, water point and urban EA boundaries. Our process starts by stratifying the country based on the administrative boundary and population types. Then, the quadtree algorithm was employed to generate the population sampling frame within each stratum using a high-resolution gridded population. Often, this algorithm is used for spatial data structure and in image compression. The approach is based on the idea of a quadtree decomposition in which an image successively subdivided into four equal size quadrants, until the content of each quadrant is homogenous. For the first time, this idea was implemented to generate a population sampling frame using a high gridded population dataset. After generating the population sampling frame, GIS and manual approaches were adopted to complete the survey sample and selection process. A flowchart of the process is given in Fig. 1.

**Fig. 2:**
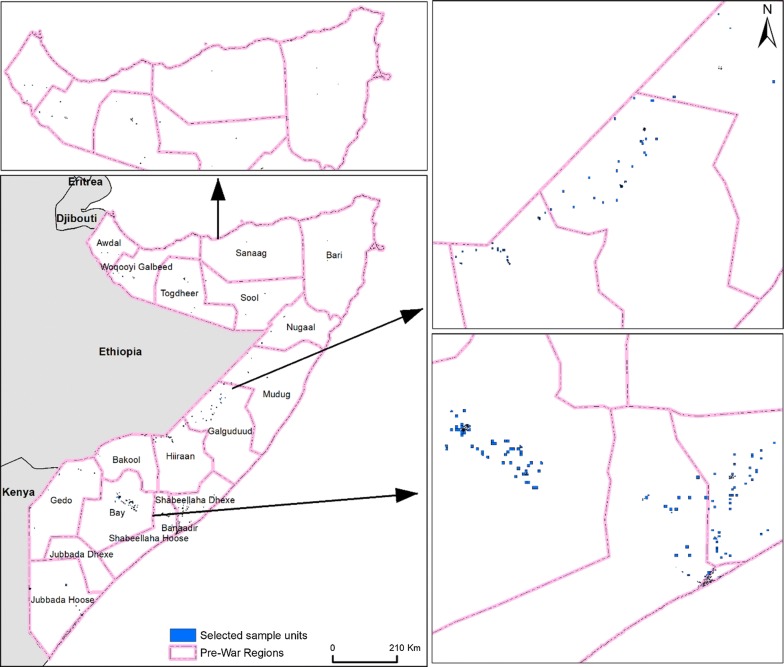
Boundaries of 18 pre-war regions in Somalia with the spatial distribution of 6384 selected samples in the secure areas

The developed methodology was designed to implement wave 2 of the Somali High-Frequency Survey [37] to better understand livelihood and vulnerabilities and, particularly to estimate national poverty indicators. The fieldwork was conducted between December 2017 and February 2018. Based on previous data and targeting estimates of consumption indicators with less than 10% relative standard errors for key sub-populations, a sample size of 6384 households was planned (Table 1, Fig. 2).

**Table 1:** Comparison of population sizes among the population sampling units for quadtree and uniform grid cell (UGC) 1 × 1 km and 3 × 3 km population sampling frame at the country scale

Statistical summary	Quadtree	Uniform grid cell	
		1 × 1 km	3 × 3 km
Minimum	0	0	0
Maximum	3498	42,384	129,132
Mean	44	16	122
Standard deviation	232	330	1648
Coefficient of variation	5	20	14

### Somalia census data

Somalia’s first national census was conducted in 1975 which published limited results [7]. The most recent census in Somalia was carried out in 1986/1987 but the results were not published officially [38]. Since then many efforts have been made by development agencies to obtain reliable population distribution data. For instance, Vaidyanathan [39] made the most notable attempt in his consultancy report and the population totals and demographic characteristics were widely accepted as the most reliable data, with a projected population for 2005 of just over seven million people. Another attempt was made by the United Nations Development Programme (UNDP) in 2005 to establish the estimated population by sex and region from 2005 to 2010 for each year. In 2014, the United Nations Population Fund (UNFPA) conducted the first extensive household survey among the Somali population in decades [7]. In this report, comprehensive and reliable population estimates by region and important demographic characteristics were provided. Based on this report the total population was estimated to be 12.3 million, of which 42% are urban, 23% rural, 26% nomadic, and 9% are internally displaced [7]. However, none of these datasets mentioned previously provide a suitable sampling frame for a national survey in terms of population counts/estimates at a fine geographic scale.

### Boundary data

The 1986 pre-war region boundaries were used to define 18 pre-war administrative regions (Fig. 2) [6]. For the purpose of this study, other boundaries were either created or obtained for urban, rural and IDP camp boundaries to independently sample different population types. IDP boundaries were created from camp locations which were available in different spatial formats (polygons, lines and points; see Additional file 1: Table S2 for the source of IDP settlements boundaries). These were harmonised to boundary polygons (see Additional file 1: Figure S2 for IDP boundary creation).

The urban area boundary was defined by previous urban enumeration areas which were used in the 2014 Population Estimation Survey of Somalia (PESS) [7]. The remaining areas outside of the urban areas and IDP camps were considered as rural areas. The overwhelming majority of Somalia is rural, with vast areas likely including nomadic peoples who account for approximately 3.2 million people in Somalia [7].

### Sample stratification

For the survey to deliver estimates for the main administrative subdivisions of the country and for sub-population types, strata were defined by the intersection of pre-war region boundaries and population types (urban, rural, nomadic and IDP) to create a total of 57 strata (see Additional file 1: Figure S1 for stratification map). Sub-populations in the urban centers of Mogadishu, Baidoa, and Kismaayo, in fisheries livelihood zones in coastal areas, and IDP host communities were of particular interest and therefore deliberately oversampled. The remaining allocation was based on a Neyman technique of optimal allocation to select PSUs in each design stratum [40], while maintaining a minimum of two PSUs per stratum to allow for the computation of stratum level variance estimates. Optimal allocation is given by:

$$n_{h} = \frac{{N_{h} S_{h} }}{{\mathop \sum \nolimits_{h = 1}^{H} N_{h} S_{h} }}$$

where $n_{h}$ is the sample size in stratum *h*, *n* is the total sample size, *H* is the total number of strata, *N*_*h*_ is the total population of stratum *h*, *N* is the total overall population, and *S*_*h*_ is the standard deviation in stratum *h*. Hence, the number of households to be interviewed per stratum is mainly determined by the variability of consumption within the stratum (*S*_*h*_). An estimate of *S*_*h*_ was derived from the results of the Somalia High-Frequency Survey SHFS Wave 1 [41]. The population size only matters for practical purposes in very small strata below 10,000 households. In the absence of a recent population census, the population of each stratum was derived from UNFPA’s Population Estimation Survey of Somalia (PESS), which contains detailed estimates for each population type and administrative unit of interest (see Additional file 1: Table S1 for summary of allocated sample units within strata).

### Gridded population dataset

**Fig. 3:**
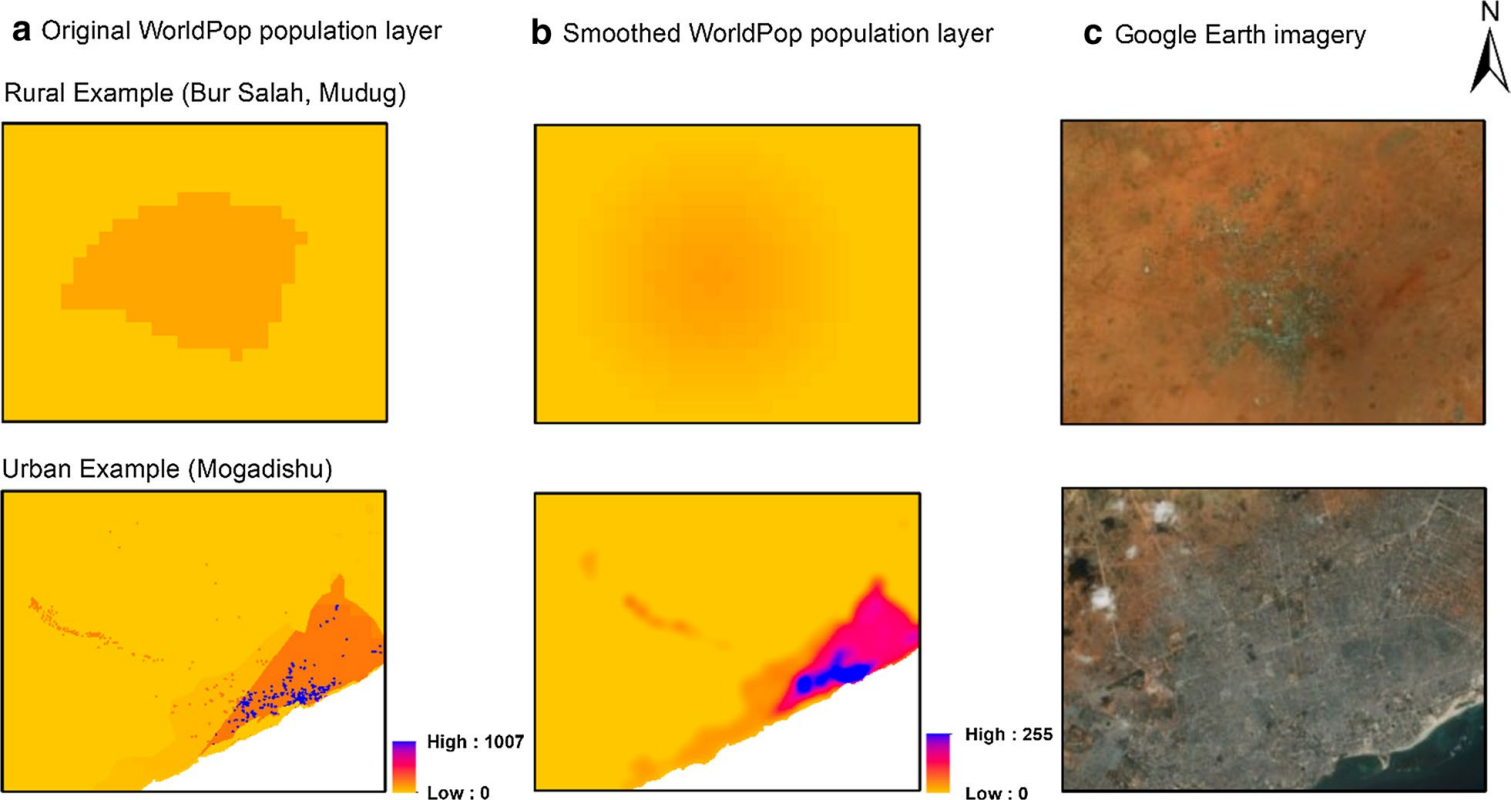
**a** Original WorldPop layer [SOM15adjv4.tif = Somalia (AGO) population count map for 2015 [15] adjusted to match UN national estimates (adj), version 4 (v4)], **b** smoothed layer after applying a Gaussian smoothing kernel technique with a standard deviation of 500 m and **c** high-resolution satellite imagery [48]

The choice of gridded population surface is important for the reliability of the underlying sampling frame. In Somalia, High-Resolution Settlement Layer (HRSL) and Demobase were not available at the time of this study. LandScan, Global Rural–Urban Mapping Project (GRUMP) and Gridded Population of the World (GPWv4) version 4 each estimate the population in 1 km × 1 km grid cells which is too large to use as a sampling unit in urban areas. Global Human Settlement Population Grid (GHS-POP) is essentially the Gridded Population of the World (GPWv4) dataset further disaggregated to 250 m × 250 m grid cells and constrained to settled areas. Settlement areas, defined by the GHS-BUILT dataset, unfortunately often omit small rural settlements and overestimate the population in urban areas [42]. The WorldPop dataset for Somalia was created under the AfriPop project based on a land cover-based model Fig. 3a) [43]. Although less accurate than the more current WorldPop Random Forest-based estimates [13], these early WorldPop estimates were still more accurate than other gridded population datasets including GPW, Global Rural–Urban Mapping Project (GRUMP) and LandScan [44]. Further, WorldPop produces estimates in 100 m × 100 m grid cells, allowing the greatest flexibility to aggregate cells into larger sampling units. Census-independent gridded population datasets are expected to become available in multiple LMICs in 2019 but were not available at the time of this work [45, 46].

Given the coarseness of the input population estimates in this model, we observed that the population might not have been distributed adequately within settlement areas. To improve this limitation and for the sake of better sampling weight calculation, a Gaussian smoothing kernel technique with a standard deviation of 500 m was applied (Fig. 3b) [47]. The original and smoothed population datasets, along with high-resolution satellite imagery can be seen in Fig. 3. To assess the impact of applying a Gaussian smoothing kernel technique with a standard deviation of 500 m on the homogeneity of the output sampling units, a comparison analysis is conducted between quadtree sampling units derived from smoothed and unsmoothed WorldPop datasets.

### Population sampling frame using a quadtree algorithm

**Fig. 4:**
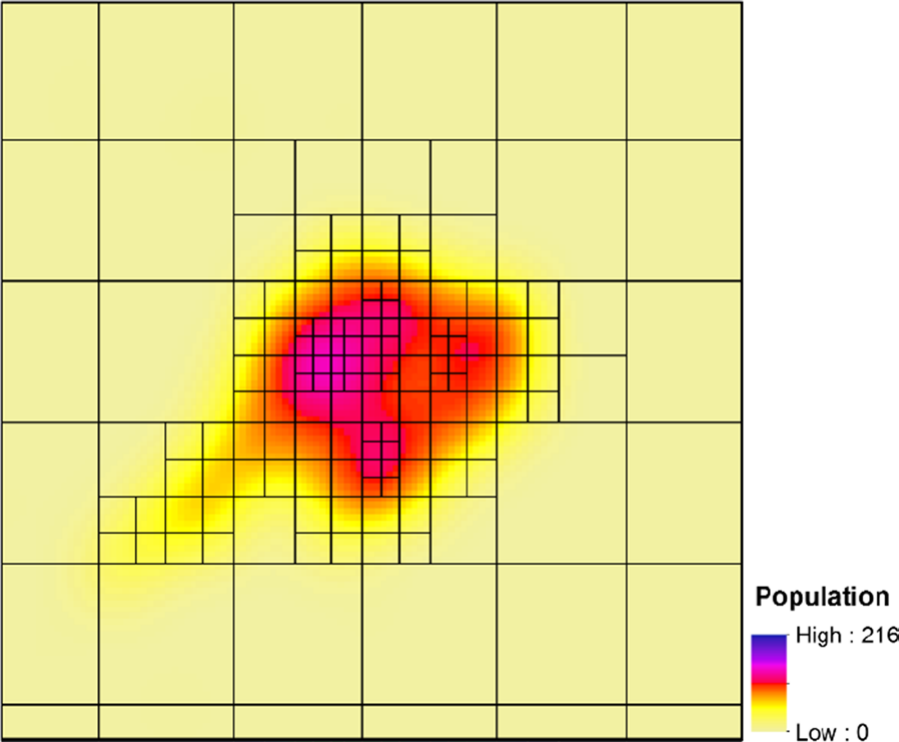
Results of the quadtree approach on smoothed WorldPop population data in Baydhabo, Somalia. Here, the maximum grid size is set to 3 × 3 km and the maximum population set to 3500

In urban areas and IDP camps, PSU boundaries were clearly defined. However, in rural areas where PSU boundaries were not predefined, we created PSUs using the quadtree algorithm by partitioning the smoothed gridded population layer into sampling units of approximately equal population. In a quadtree algorithm, each internal node in the underlying tree has exactly four children [36]. This approach is commonly employed to partition a two-dimensional space by recursively decomposing it into four equal quadrants or regions [49]. The approach splits an area into successively smaller quadratures by checking to see whether the content of each split meets a prescribed value. The output could be rectangular or square or may have arbitrary shapes. A quadtree algorithm can categorize many types of data including points, lines and regions [49]. The entire area of rural strata was divided into square grid cells using a quadtree algorithm. In this case, the population map was used as the unit of measure and was split successively until each square had a population of less than 3500 (Fig. 4). As well we restricted cell sizes to have a maximum geographical area of 3 × 3 km to keep enumeration areas manageable in size for field teams. The result was a spatially complete set of sampling units with smaller units in areas of dense population. The quadtree algorithm was implemented in R [50] and the code are provided in Additional file 1: Section 2 for Quadtree R based code.

### Sampling strategy

The sampling frame for this survey is the exhaustive list of sampling units for every stage in the multi-stage selection process (e.g. PSU, Secondary Sampling Unit (SSU), Tertiary Sampling Unit (TSU) and so on). Multistage sampling is employed near universally for household surveys, particularly as a cost control strategy where the population is sparsely distributed [51]. For the current work, each stratum has a list of sampling units. Each sampling unit must have an estimated population to derive a selection probability as the estimated population divided by the total population of the stratum. Nomadic populations were not included in this sampling frame due to their lack of a permanent place of residence, and are treated in a separate piece of work.

#### Selection of PSUs and first-stage sample selection

The quadtree algorithm was used to derive PSUs such that each PSU has a known area and estimated population. Sample selection was with-replacement and based on a systematic random sampling technique with selection probability proportional to size (PPS). Assuming accurate population estimates, in PPS random sampling, each unit’s probability of selection is proportional to its population (Probability > 0). In addition, in each stratum with an additional 20% replacement sample was selected in case the main PSUs needed to be replaced due to the absent population. While replacements generally lead to bias in estimates, replacements in the case of no population found do not cause bias because the empty PSU is determined to be out-of-scope. It is, however, not possible to calculate accurate probability weights for replacement households as the replacement PSU must be assumed to have the same probability of selection as the original, even though it has a different population. All selected PSUs were visually checked on current high-resolution imagery basemap [48], and PSUs with no visible structures were substituted with a randomly selected replacement PSU.

#### Creation of SSUs and third stage sample selection

**Fig. 5:**
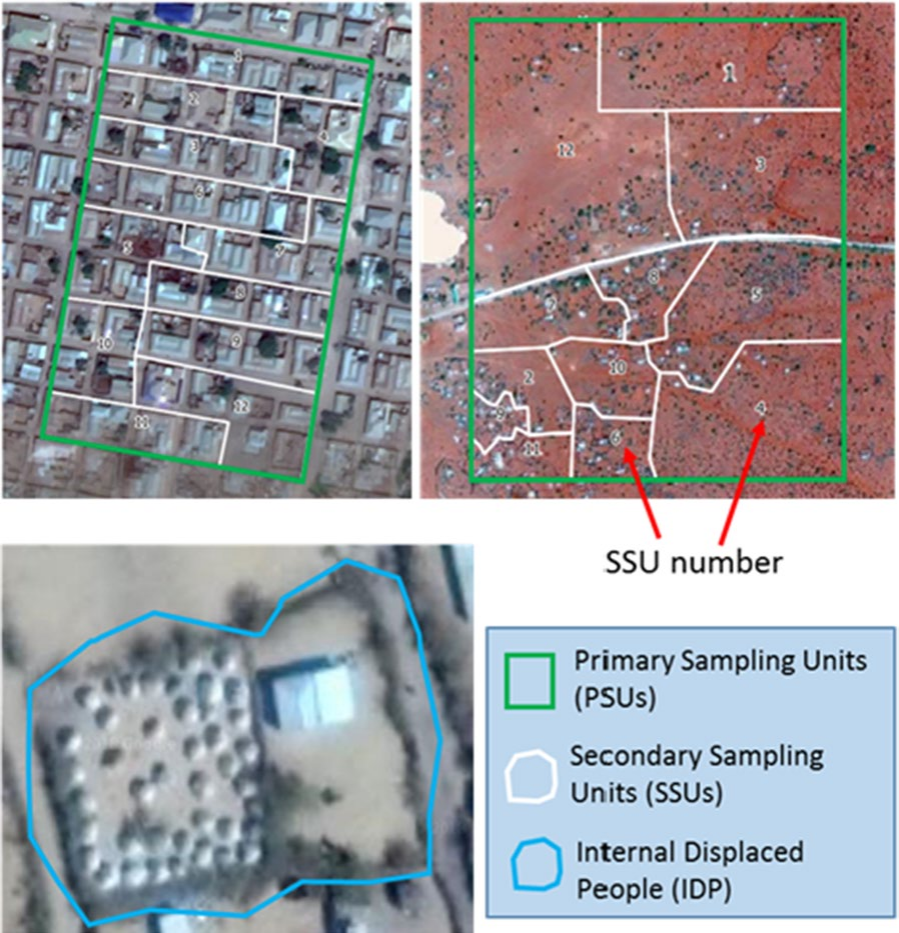
Outlines of different sample unit boundaries such as Primary Sampling Units (PSU), Secondary Sampling Units (SSU) and Internally Displaced People (IDP) on high-resolution satellite imagery [48]

The selected PSUs were manually segmented into 12 approximately equal-sized SSUs each (Fig. 5). We manually delineated SSUs, using current high-resolution satellite imagery from the Esri satellite imagery basemap [48] by counting the number of visible structures and taking account of natural boundaries. Each SSU contained 1 to 12 structures visible from a central point on the ground. For several special cases, three criteria were employed: (i) PSU containing less than 12 structures were not segmented, (ii) in PSUs containing more than 150 structures, more than 12 SSUs were delineated with 12 buildings each, (iii) in very large PSUs that were selected two or three times in a stratum with a short list of sampling units, the number of delineated SSU was scaled up proportionately to the number of times the PSU was selected.

SSUs were selected with equal probability from a household list generated by enumerators canvasing the selected areas to produce a list of all residential structures, and one structure was randomly selected in each SSU [52]. At the structure, the enumerator recorded the number of households, and the tablet again randomly selected one household in the structure to be interviewed. One household per SSU was used to minimize potential loss of efficiency due to homogeneity of the variables of interest within SSUs. In the general case of 12 SSUs per PSU, a total of 12 households were selected for an interview with one selected household in each SSUs, though the number of selected households per PSU spanned from 1 to 36 depending on the number of SSUs due to multiple selections in strata with small populations (see Additional file 1: Section 3 for step by step guide to creating field maps).

### Comparison of sampling units

An ideal sampling frame is comprised of sampling units of approximately equal population size within a given stratum to minimize variation in the sampling weights. Commonly, the survey practitioner creates UGC which sometimes consists of a single cell or a group of smaller cells (e.g. 3 × 3 km) to generate the first primary sampling units. This subdivision of the country does not consider the population density and area which might have an impact on the degree of population homogeneity and spatial variation within the sampling units in a given region. Therefore, we measure the degree of population homogeneity and spatial population variation size within the created sampling units by calculating standard deviation (std), which is a measure that is used to quantify the amount of variation of a set of values to the mean, and coefficient of variation (cv) at country and regional scale for both quadtree and UGC 1 × 1 km and 3 × 3 km approaches.

## Results

### Unsmoothed vs smoothed WorldPop data to generate QT sampling frame

**Fig. 6:**
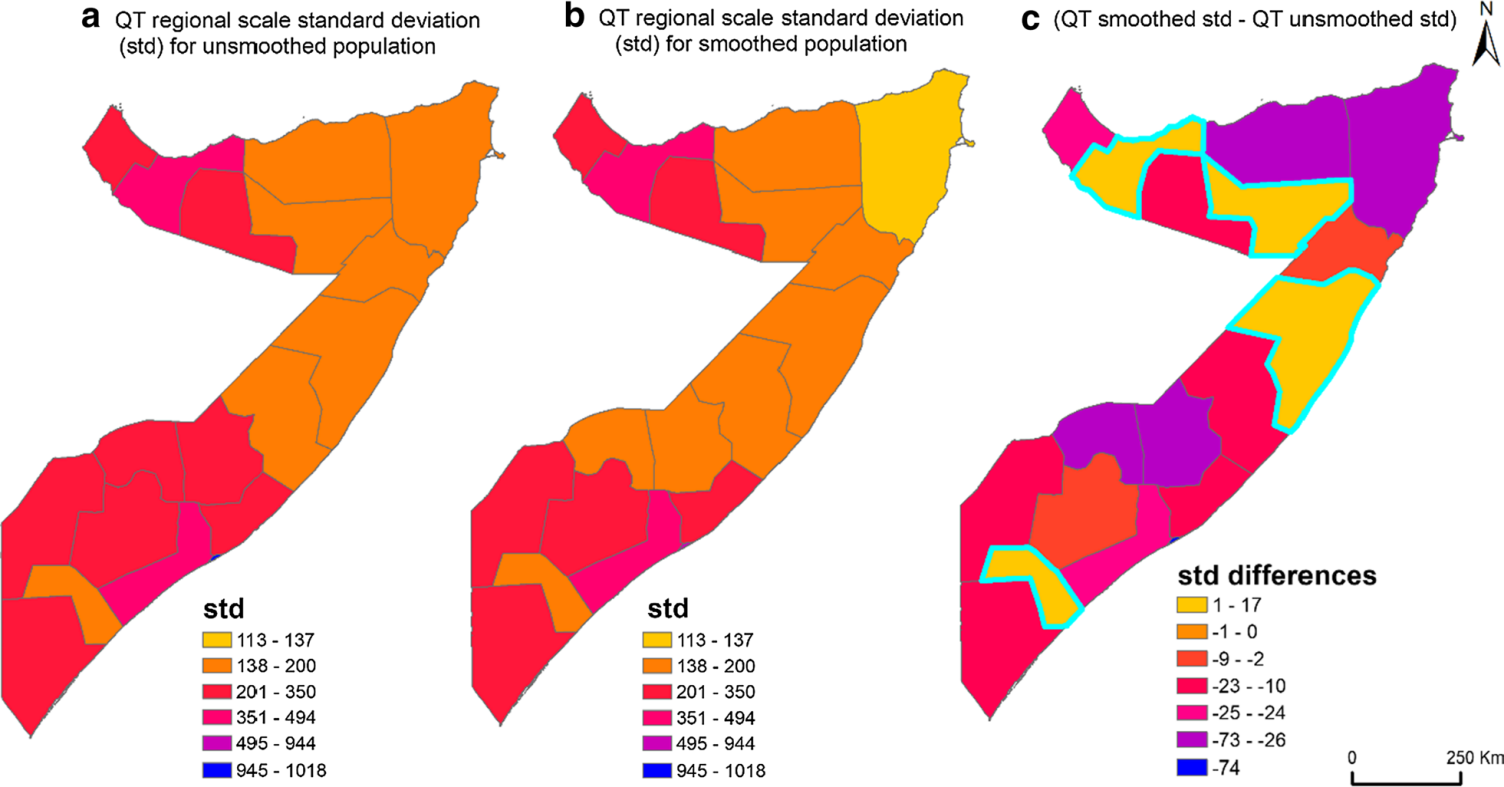
Shows the difference between quadtree regional standard deviation where **a** unsmoothed WorldPop data, **b** smoothed WorldPop data were used to create population sampling frame and **c** the difference between the standard deviation of both datasets

Figure 6 shows the impact of applying a Gaussian smoothing kernel technique with a standard deviation of 500 m on the homogeneity of the output sampling units within the 18 pre-war regions using the QT approach. It can be seen from the Fig. 6c that using the smoothed WorldPop data has improved population homogeneity within the regional sampling units in the majority of the pre-war regions except Woqooyi Galbeed, Sool, Jubbada Dhexe and Mudug (std = 17, 12, 6, 3). The highest population homogeneity improvement within the output sampling units was observed in Banadir (std = − 74).

### Survey sampling units

**Fig. 7:**
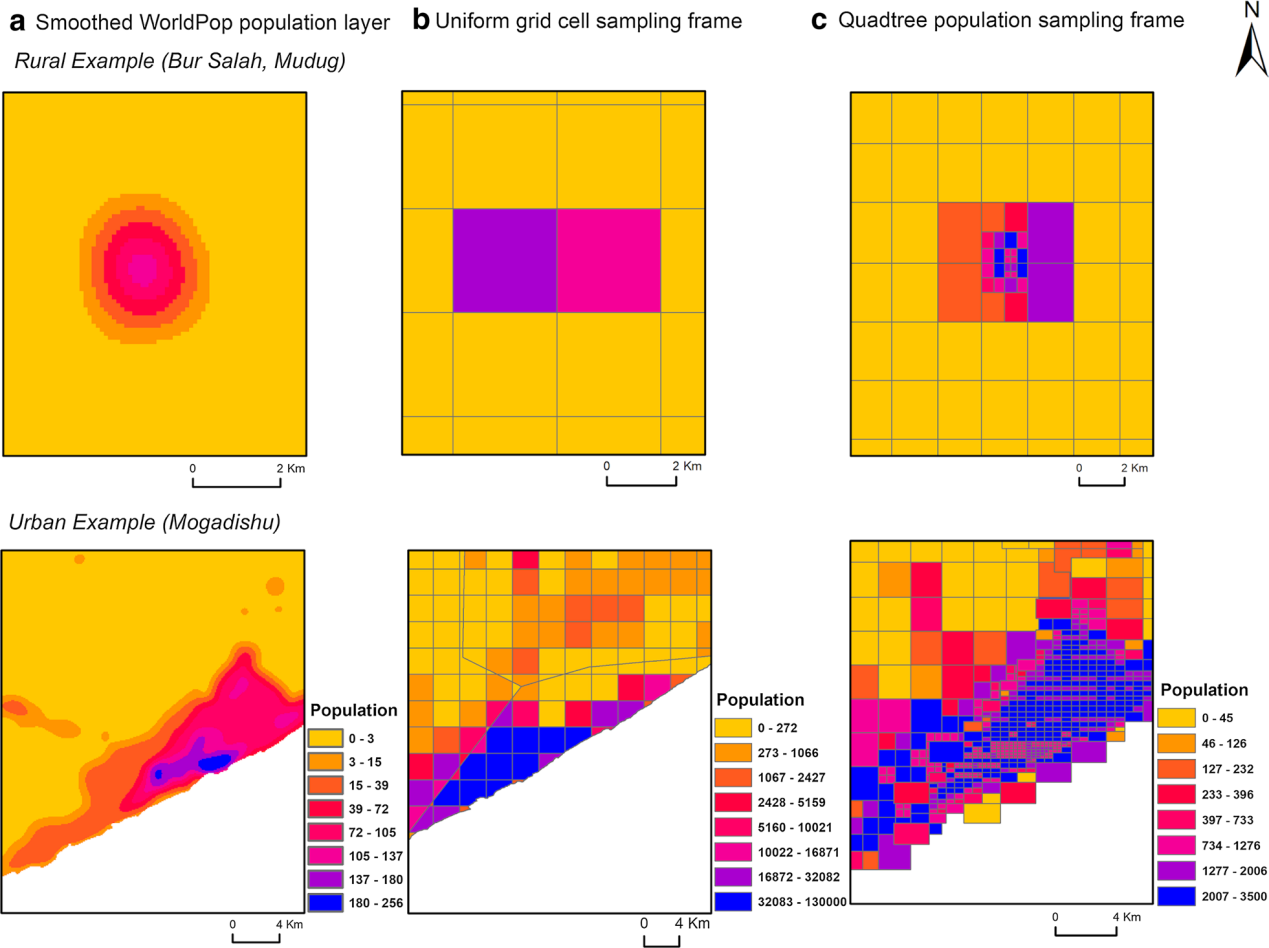
Examples of **a** smoothed population surfaces (**b**) and primary uniform grid sampling units (3 × 3 km) (**c**) after applying the quadtree algorithm

The quadtree approach used here produced much more heterogeneous sampling units than the UGC (3 × 3 km) approach (Fig. 7). The quadtree algorithm resulted in PSUs with population sizes ranging from 0 to 3500. By contrast, the UGC approach created PSUs with population sizes ranging from 0 to 42,400 for 1 × 1 km and 0 to 130,000 for 3 × 3 km. The 2014 Population Estimation Survey of Somalia (PESS) resulted in 7210 urban PSUs [6], and from various sources, we defined 500 IDP PSUs.

**Fig. 8:**
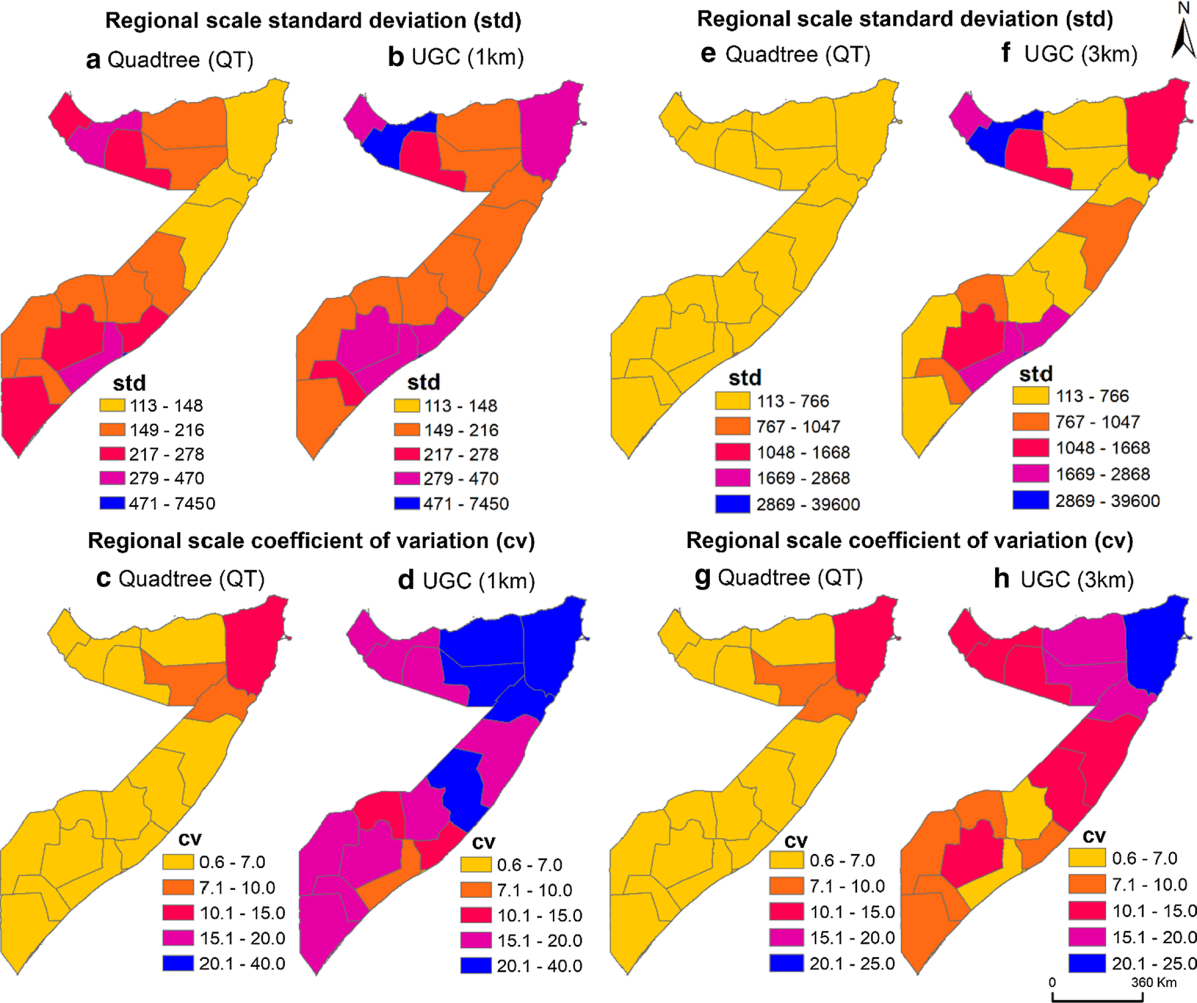
Population homogeneity of the output sampling units within the 18 pre-war regions produced by quadtree and uniform grid cell (UGC) 1 × 1 km and 3 × 3 km approaches **a**, **e** quadtree regional scale standard deviation (std), **b** UGC 1 × 1 km 18 pre-war regional scale standard deviation, **c**, **g** quadtree 18 pre-war regional scale coefficient of variation, **d** UGC 1 × 1 km 18 pre-war regional scale coefficient of variation, **f** UGC 3 × 3 km 18 pre-war regional scale standard deviation, **h** UGC 3 × 3 km 18 pre-war regional scale coefficient of variation

**Fig. 9:**
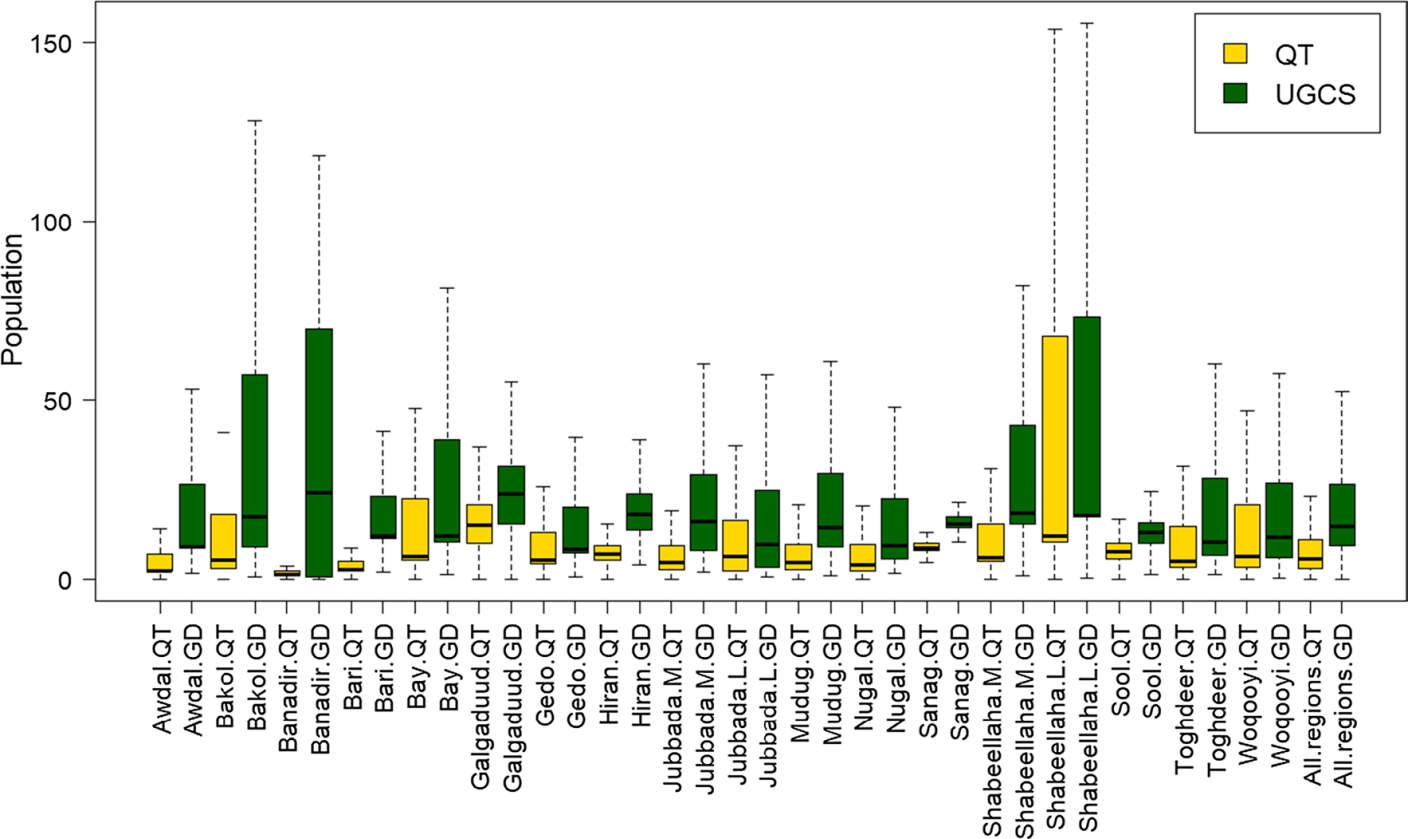
Box plot of population size variations among sampling units in the 18 pre-war regions using the uniform grid cell approach (UGC 3 × 3 k) and quadtree approaches. Within each box, horizontal black lines denote median values; boxes extend from the first quartile and third quartile of each group’s distribution of values; vertical extending lines denote adjacent values. Note that the value for the Banadir region was divided by 1000 in both quadtree and UGC 3 × 3 km

Nationally, population size within PSUs was relatively homogeneous using quadtree (std = 232 for quadtree vs 330 and 1648 for UGC 1 × 1 km and UGC 3km) with a lower coefficient of variance (cv = 5 for quadtree versus 20 and 14 for UGC 1km and UGC 3 × 3 km) (Table 2). In pre-war regions, the quadtree results also produced PSUs with more homogenous standard deviation (std) (113 < std > 1047) (Fig. 8a, e) whereas UGC had much greater range of variations [1 × 1 km (113 < std 7450), 3 × 3 km (113 < std > 3952)] (Fig. 8b, f). Similarly, this difference can be seen in coefficient of variation comparison maps in which coefficient of variation ranged from 1 to 11 for quadtree (Fig. 8c, g) but 1 to 40 for UGC 1 × 1 km (Fig. 8d) and 1 to 25 for UGC 3 × 3 km (Fig. 8h). Figure 9 summarizes these results in a boxplot showing greater variability in PSU population size in quadtree pre-war regions results compare to the UGC 3x3km.

**Table 2:** Population by age and gender based on Somalia High frequency surveys in 2017/2018

	Female (%)	Male (%)	Total (%)
0–14 years	23.67	25.37	49.05
15–34 years	16.91	13.18	30.09
35–64 years	8.47	10.91	19.38
65+ years	0.58	0.90	1.48
Total	49.64	50.36	100.00

### Summary of field survey results

The methodology presented here was used to design the survey strategy for Wave 2 of the Somalia High-Frequency Survey which was conducted between December 2017 and February 2018, collecting data from 6384 households representing urban, rural, displaced and nomadic populations [37]. The results from the survey show that the Somali population is predominantly young, with close to half of Somalis younger than 15 years old (Table 2), while the ratio women to men in the population is approximately even. Only 42% of households are headed by women (Table 2). In line with a very young population, the mean and median household size is large at 5.37 and 5, respectively, and a dependency ratio of 1.3 (Table 3). The poverty rate in areas that the survey could access is 69% and poverty rates in urban households (64%) are lower than rural (72%), IDP (76%), and nomadic households (72%) (Table 3). Almost 80% of households have access to improved sources of water and have literate household heads, while less than 50% have access to improved sanitation [37, 53].

**Table 3:** Household demographics and socio-economic characteristics based on Somalia High frequency surveys in 2017/2018

Population type	Overall	Urban	Rural	IDP	Nomads
Household size (mean)	5.37	4.97	5.51	5.41	5.85
Household size (median)	5	5	5	5	6
Household head male	58%	49%	63%	46%	77%
Dependency ratio	1.30	1.09	1.51	1.40	1.42
Poverty rate (US$ 1.90 international poverty line)	69%	64%	72%	76%	72%
Household head literate	77%	84%	68%	85%	67%
Improved water access	77%	84%	68%	85%	67%
Improved sanitation access	46%	70%	40%	51%	8%

### Sampling weight calculations and implication for precision

The sampling weights are based in the inverse probability of selection. Following the sample selection methodology described in “Sample stratification” section, the probability of selection of PSU i in stratum h is $P_{1} = \frac{{n_{h} k}}{{N_{h} }}$, where k is the number of PSUs selected in that particular stratum. In the majority of the cases where one household was selected from each of the 12 approximately equal sized second stage units, the probability of selection of an individual household is $P_{2} = \frac{1}{{m_{h} }}$, where $m_{hi} \cong \frac{{n_{hi} }}{12}$. Appropriate adjustments to the weight calculations were made for the three special cases described in “Creation of SSUs and third stage sample selection” section.

One consequence of using a gridded population approach over a traditional design is that the variation in the size of the PSUs leads to high levels of variation in the weights [54]. To illustrate the implications, we compared the efficiency of the Somali High-Frequency Survey design with a subset of the 2015/16 Kenya Integrated Household Budget Survey (KIHBS), was selected from a census-based national sampling frame. The full KIHBS dataset has more than 21,000 observations, but we limit our analysis to 14 counties that are most similar to Somalia (Mombasa, Kwale, Kilifi, Tana River, Lamu, Taita Taveta, Marsabit, Isiolo, Meru, Tharaka Nithi, Embu, Kitui, Machakos, and Makueni). This subsample includes 6312 observations in 688 unique clusters, with an average cluster size of 9.2. The Somalia survey has 6092 observations in 413 unique clusters, with an average cluster size of 14.8. The weight variation produced in the final sampling weights for the Somalia survey is extreme, with the highest weight being more than 85,000 times larger than the smallest weight, compared with 423 times in the KIHBS. This finding exists despite the fact that the KIHBS itself is considered to have high variation—the similarly sample sized 2018 Sierra Leone Integrated Household Survey (SLIHS) has a difference in weight values of only 23 times. The normalized variance of the sampling weights in Somalia is 6482, compared with 431 in the KIHBS and 68 in the SLIHS.

The high variance in the weights reduces the precision of the estimates. This concept can be illustrated by the design effect, which measures the “penalty” paid by using a complex sample design over a simple random sample. The design effect for a key variable of interest, per capita household expenditure, in the Somalia survey is 5.5 compared to 4.6 in the KIHBS subsample. This difference, however, is partially attributable to the larger cluster sizes in the Somalia survey, which lead to higher design effects. To isolate the impact of the unequal weights, we compare only that component of the design effects [55]. The unequal weight effect in the Somalia survey is 3.2 compared with 2.1 on the KIHBS, which translates into confidence intervals that are more than 50% wider in the Somalia design compared to a more traditional approach. It may be possible to some extent to mitigate this issue by windsorizing the sampling weights, but inefficiencies will likely remain.

## Discussion

We have described the first usage of a quadtree algorithm approach to derive a population sampling frame from gridded population data. Prior studies have noted the importance of the quadtree algorithm for various purposes such as spatial data dimension reduction, computer vision and image segmentation [56, 57]. This approach can be used to generate a viable population sampling frame for any year where a high-resolution gridded population dataset is available. This is crucial in countries where population and national enumeration area boundaries are outdated, incomplete or unavailable (e.g. DR Congo, South Sudan, Nigeria, Iraq and Afghanistan). For instance, in Somalia, EAs are only available for urban areas which cover only ~ 1% of the country [7]. Therefore, sophisticated approaches are in need to fill this gap.

Previously, UGCs have been used to create the population sampling frame in order to select the PSUs. For instance [22], used first-stage sampling units with a resolution of 2700 × 2700 m. In some areas, these large cells may correspond with high-density population areas which then need substantial time, cost and human resources to segment into manageable SSUs. For example, in the current example of the work presented here, a total population for a sample unit in Somalia could reach up to 130,000 and 42,400 people using the UGC 3 × 3 km and 1 × 1 km approaches (Fig. 7). In other areas, the sampling units could be small with just a few households, increasing the number of clusters needed to reach the minimum sample size and making data collection more expensive. In addition, UGC with large areas in a dense population is not feasible for enumeration or monitoring. By contrast, using a quadtree approach enabled us to tune the population and area constraints based on our requirements, resulting in a user-friendly sampling frame with much-improved population homogeneity within the population sampling units. The greater similarity of the cluster in terms of the population provides a more equal probability of selection, increasing the efficiency of the design and decreasing overall survey costs. This approach has also the advantage over other gridded population sampling approaches in which grid cells were selected with PPS then PSUs were “grown” by adding neighbouring grid cells after selection [11, 32] as our approach produces sampling units with preferable population size and area before carrying out the sample selection.

The quadtree algorithm relies on gridded population data to generate the population sampling frame as well as data on uncrossable boundaries and design stratifications (e.g. administrative boundaries, settlement type, agroecological zone, etc.). The quality of these source data will, therefore, have an impact on the quality of the generated population sampling frame. Of the available gridded population datasets, this work used the WorldPop layer due to its high resolution, accuracy, better spatial distribution and robust modelling methodology compared to other available gridded population datasets [43]. However, using the projected population dataset based on the old census, limited coverage of some covariates used in the modelling to disaggregate the population, vast unsettled area with a non-zero probability of population and highly mobile Somali population mean that improvements could be made to the underlying population datasets for Somalia. In addition, in the original WorldPop layer, the population values were not always distributed adequately within the settled areas. This could be due to the settlement growth or lack of quality in the input data. For instance, a village might have only a few pixels with a high population number creating a sharp contrast, although its coverage area is larger and the transition from sparse to dense population is more progressive. Our usage of a Gaussian smoothing kernel technique with a standard deviation of 500 m resulted in a more adequately distributed population surrounding a high-density pixel while preserving close to the total population count in the area. For instance, applying the smoothing technique on the population data has improved the homogeneity within the majority of the pre-war regions, particularly at the urban area of Banadir. However, this approach was not based on any additional data and other approaches could provide better population estimates. It is worth noting that WorldPop has released a new version of the population data on the global scale from 2000 to 2020 in which random forest model and other new techniques were incorporated resulted in much improvement in the previous concerns [12, 13, 58].

Outdated census data could lead to coverage errors in the sample frame. Although gridded population datasets can improve older census datasets by re-distributing population to areas of the new settlement, the sample frame may contain inaccuracies if the population distribution changed substantially since the last census due to urbanization, differential urban/rural fertility and mortality rates, or displacement due to natural- and human-caused disasters. Areas with greater population growth will be under-represented in the sample weights. The WorldPop Landcover model is not as accurate as of the WorldPop Random Forrest model by assuming similar counts of people per pixel within each landcover type; thus, variability in the sample frame may have been improved using the Gaussian smoothing technique. Future application of the quadtree should adopt more recent high gridded population data [12].

A strength of this sampling approach was that the use of a probability-based household selection protocol (a) required only one visit to the field, (b) allowed accurate calculation of sampling probability weights, and (c) prevented enumerators from having to make household selection decision in the field. In Somalia, armed conflict and frequent occurrence of droughts pose security risks for field staff [59]. To ensure the safety of field staff, our approach removed the requirement for a separate household mapping and listing activity before the selection of households and interviews. The creation of SSUs and counting of buildings provided a basis for the robust calculation of household sampling weights after the survey. Further, the random number generator on the tablet ensured that enumerators did not make decisions about the household selection in the field, and therefore minimized potential selection bias. Intentional or unintentional selection bias is a major threat in survey protocols such as a random walk or spin-the-pen which leaves household selection decisions with the enumerator in the field [60, 61].

## Conclusion

Gridded population datasets are increasingly used to create a population sampling frame in the absence of census data. However, previously used approaches were limited in their ability to create sampling units of similar population size before sampling. We have introduced an alternative method by using the quadtree algorithm for the first time to create sampling units of approximately equal population. The algorithm can derive the sampling units based on prior constraints such as population and area which is more cost and time-efficient compared to the UGC approaches. In addition, the algorithm creates the sampling units based on user requirements prior to the sample selection which solves the incorrect probability of selection in growing the grid samples to a given size [32]. It considers the population distribution in creating the sampling units in rural and urban settings rather than creating uniform sampling units [22, 33]. Furthermore, it avoids creating complex and snaky shapes (always square). In terms of homogeneity, the standard deviation and coefficient of variation for the sampling units were produced by quadtree is much smaller than the UGC approaches. The method also does not need strong computation power and minimal user interaction is required. Overall, the strategy has the potential to improve surveys in high-risk environments by reducing the number of field visits and potential use in wider contexts.

## Supplementary information

**Additional file 1: Figure S1.** Stratification map. **Table S1.** Summary of allocated sample units within strata. **Table S2.** Source of IDP settlements boundaries. **Figure S2.** IDP boundary creation. **Section 2.** Quadtree R based code. **Section 3.** Field map creation.

## Data Availability

Not applicable.
