# Supplementary material for: Using gridded population and quadtree sampling units to support survey sample design in low-income settings
Source: Int J Health Geogr. 2020 Mar 26;19:10. doi: 10.1186/s12942-020-00205-5 (PMC7099787; doi:10.1186/s12942-020-00205-5)
Supplement: Supplementary file 1 — Additional file 1: Figure S1. Stratification map. Table S1. Summary of allocated sample units within strata. Table S2. Source of IDP settlements boundaries. Figure S2. IDP boundary creation. Section 2. Quadtree R based code. Section 3. Field map creation. [file 12942_2020_205_MOESM1_ESM.docx]

**Additional file**

**Using gridded population and Quadtree sampling units to support survey sample design in low income settings**

Sarchil Hama Qader; Véronique Lefebvre, Andy J. Tatem, Utz Pape, Warren Jochem, Kristen Himelein, Amy Nieman, Philip Wolburg, Gonzalo Nunez-Chaim, Linus Bengtsson, Tomas Bird.

1. ***Strata boundaries***


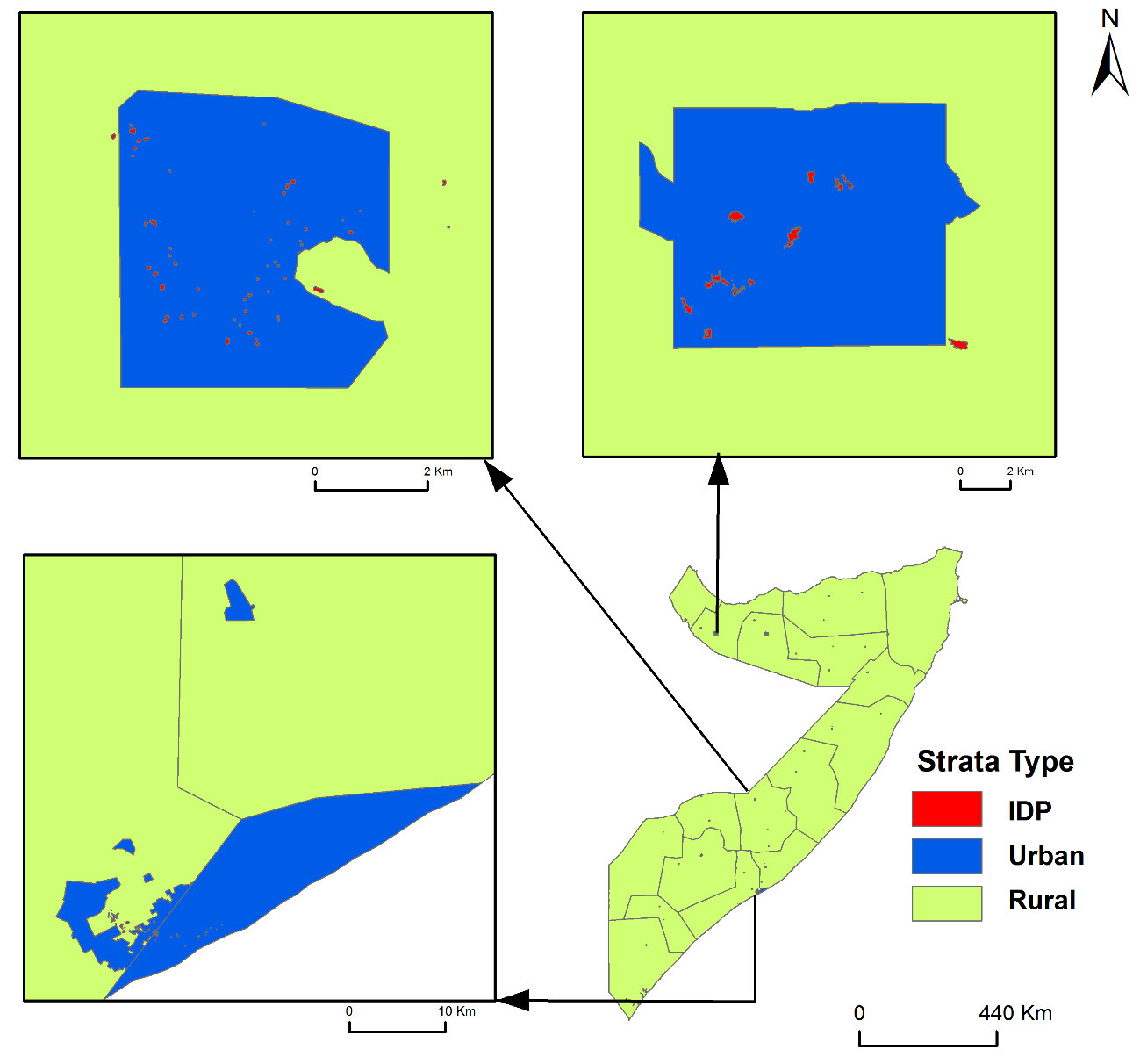


Figure S1. Map of urban, rural and internally displaced people (IDP) strata boundaries. Enlargements show typical distributions of urban, rural and IDP areas in three areas including Hiran (Belet Weyne), Hargeysa and Mogadishu.

Table S1. Summary of allocated sample units within strata


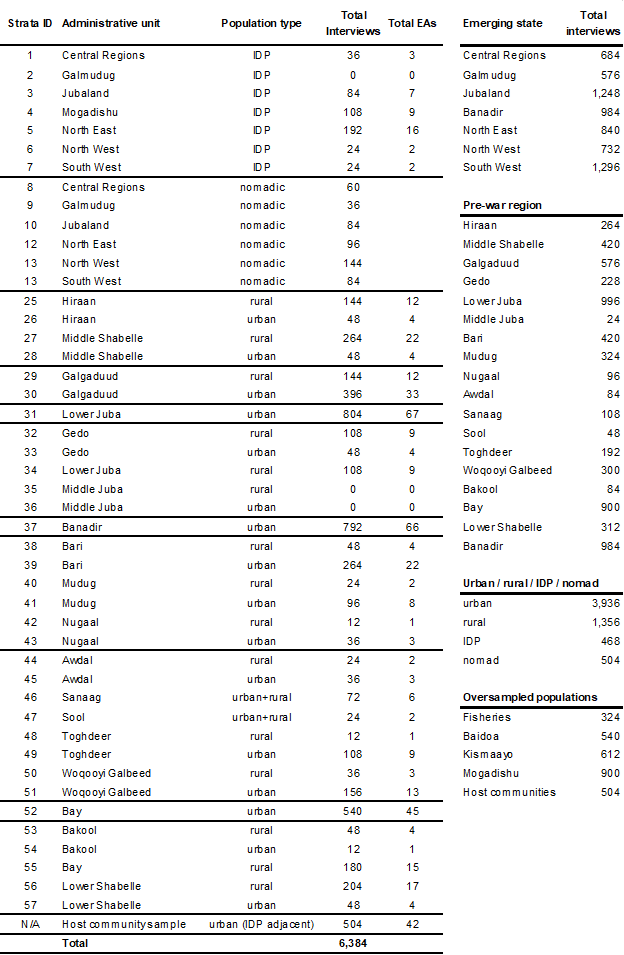


Table S2. Source of IDP settlement boundaries


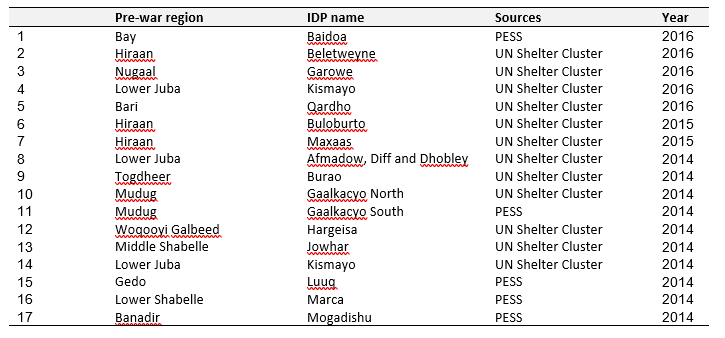


The following steps were taken to harmonise the IDP data to boundary polygons

- The original data was converted from kml file to shapefile
- Since the polygons were very close, a 10 m buffer was applied to the polygons to smooth and merge the boundaries.
- Lines were converted to polygons
- Point clusters were converted to polygons where all points within 50m were grouped. Then a 10m buffer was added to each shape to smooth boundaries (Figure 1).
- finally, “doughnut holes” were removed by combining areas within 100m of each other and removing holes smaller than 10000 square metres (Figure 1).


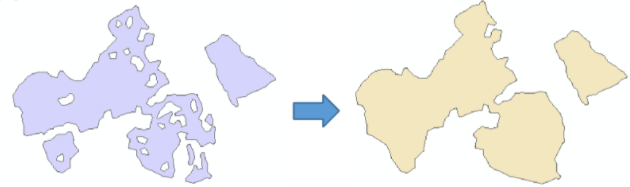


Figure S2. Visualization of methods to generate IDP camp polygons from points

1. **Quadtree R based code**

## Quadtree-based decomposition of rasters (gridded population dataset)

## Required packages

install.packages("raster")

install.packages("maptools")

install.packages("rgdal")

install.packages("sp")

install.packages("doSNOW")

## Set directory

setwd("C:/")

## Define the raster data (Gridded population data)

RasataData=raster("Raster data")

## Define the shape file to identfy the adminstrative regions or settelment types.

reg=readOGR("shape file")

RasterData.Region1=subset(reg, ID=="First Region name")

RasterData.Region1=subset(reg, grepl("Second Region name", ID))

Awdal.rast=crop(som, som.Awdal)

Banaadir.rast=crop(som, som.Banaadir)

## Regions to process

provs <- as.character(reg$ID)

## help function: split images into quads

splitim <- function(im){

r <- nrow(im)

c <- ncol(im)

dr <- round(r/2)

dc <- round(c/2)

quads <- list()

quads[[1]] <- im[1:dr, 1:dc, drop=FALSE]

quads[[2]] <- im[(dr+1):r, 1:dc, drop=FALSE]

quads[[3]] <- im[(dr+1):r, (dc+1):c, drop=FALSE]

quads[[4]] <- im[1:dr, (dc+1):c, drop=FALSE]

return(quads)

}

## **Pre-define parameters for population size and area**

minSize <- 3500 # Maximum population size

gridSize <- 32 # maximum grid dimension

### main loop

for(p in provs[1:18]){

print(p)

grid_c<- crop(som, subset(reg, ID==p))

grid<-mask(grid_c, subset(reg, ID==p))

#grid=mask(grid, UR)

dim(grid)

iml <- list() # store the list of images to process

iml[[1]] <- grid # first in the image list is the full grid

res <- FALSE # results, start false to begin with a split

res2 <- FALSE

imS <- list() # output list of split grids

# main processing loop

while (prod(res)==0 | prod(res2)==0) {

iml <- unlist(lapply(iml, splitim))

res <- unlist(lapply(iml, function(i){ cellStats(i, stat='sum') < minSize } ))

res2 <- unlist(lapply(iml, function(i){ max(dim(i)) < gridSize } ))

imS <- c(imS, iml[which(res==1 & res2==1)])

iml <- iml[which(res==0 | res2==0)]

}

## drop subscenes with no data

keep <- lapply(1:length(imS), function(i){ cellStats(imS[[i]],'mean')>0 & !is.nan(cellStats(imS[[i]], 'mean')) })

imS <- imS[unlist(keep)==T]

## output split grids

if(!dir.exists(paste0( "subscenes"))){dir.create(paste0( "subscenes"))}

if(!dir.exists(paste0( "subscenes/", p,"/"))){dir.create(paste0( "subscenes/", p,"/"))}

## output extent polygons

plylist <- lapply(1:length(imS),

function(i){

if(!is.null(imS[[i]])){

pop=cellStats(imS[[i]], sum)

e <- extent(imS[[i]])

sp <- as(e, 'SpatialPolygons')

crs(sp) <- crs(imS[[i]])

polydf <- SpatialPolygonsDataFrame(sp, data=data.frame(id=i, pop=pop))

return(polydf)}

})

## merge all to a single feature

joinpoly <- do.call(bind, plylist)

if(!dir.exists(paste0( "subshapes"))){dir.create(paste0( "subshapes"))}

if(!dir.exists(paste0( "subshapes/", p,"/"))){dir.create(paste0( "subshapes/", p,"/"))}

shapefile(joinpoly, paste0( "subshapes/", p, "/", p, "_ply_all3.shp"), overwrite=T)

pdf(paste0( "subshapes/", p, "/", p, "_plot.pdf"))

plot(grid, main=p)

plot(joinpoly, add=T)

plot(reg, add=T)

dev.off()

}

1. **Field map creation**

In the census cartography or any type of survey, detailed fieldwork maps are necessary to guide the fieldworker on the ground. Depending on the size of the survey or if it is a census, hundreds to several thousand EAs need to be mapped. The process of creating the field maps should meet two main criteria. First, since creating these maps manually can take a long time and updating them on a regular basis is also time consuming, a fully automatic generation is required. Second, the maps must be clear and have all the necessary information for the fieldworker on the ground. In addition, since tablets are used in most of the recent surveys for collecting the data, digital maps are the only compatible formats for these devices.

Here we have used automating map creating print composer atlas in free licence QGIS (<http://www.qgis.org>). The comprehensive freely available spatial information datasets from OpenStrrt Maps (OSM) could serve as the main source of the data. The following datasets were used to create the field maps:

- Pre-war region boundaries (UNDP)
- PSUs and SSUs IDs and boundaries
- Road types (OSM)
- Point of interest (OSM)
- Bar code (ORExplore.com)
- GPS reference points

The following steps were undertaken to generate the final field maps:

1. The unique bar code for each PSU was generated on an image format. This can be read easily using freely phone bar code application.
2. Since several datasets were involved, a uniform index number were computed across all the datasets to facilitate the condition statement (Index).
3. To avoid confusion and keeping only one PSU on the map, the untargeted PSUs must be turned off:

*Turning on: “Index”= @atlas featuerid*

*Turning off: “Index” != @atlas featuerid*

1. The same condition (iii) must be applied to the label.
2. For each PSU, at least two GPS points were generated. The *X* and *Y* for each point were presented on the map. The *X* and *Y* were obtained for each point using the following expression:

*concat( round($x,5),',',round($y,5)) (Note:5 means, 5 precisions)*

1. All the map keys were inserted into the maps with an automation process based on referred attribute column.
2. The final output can be named as required and the following expression was used:

*'Text'|| "Column Name" || 'Seperation' || 'Text' || "Column Name".*


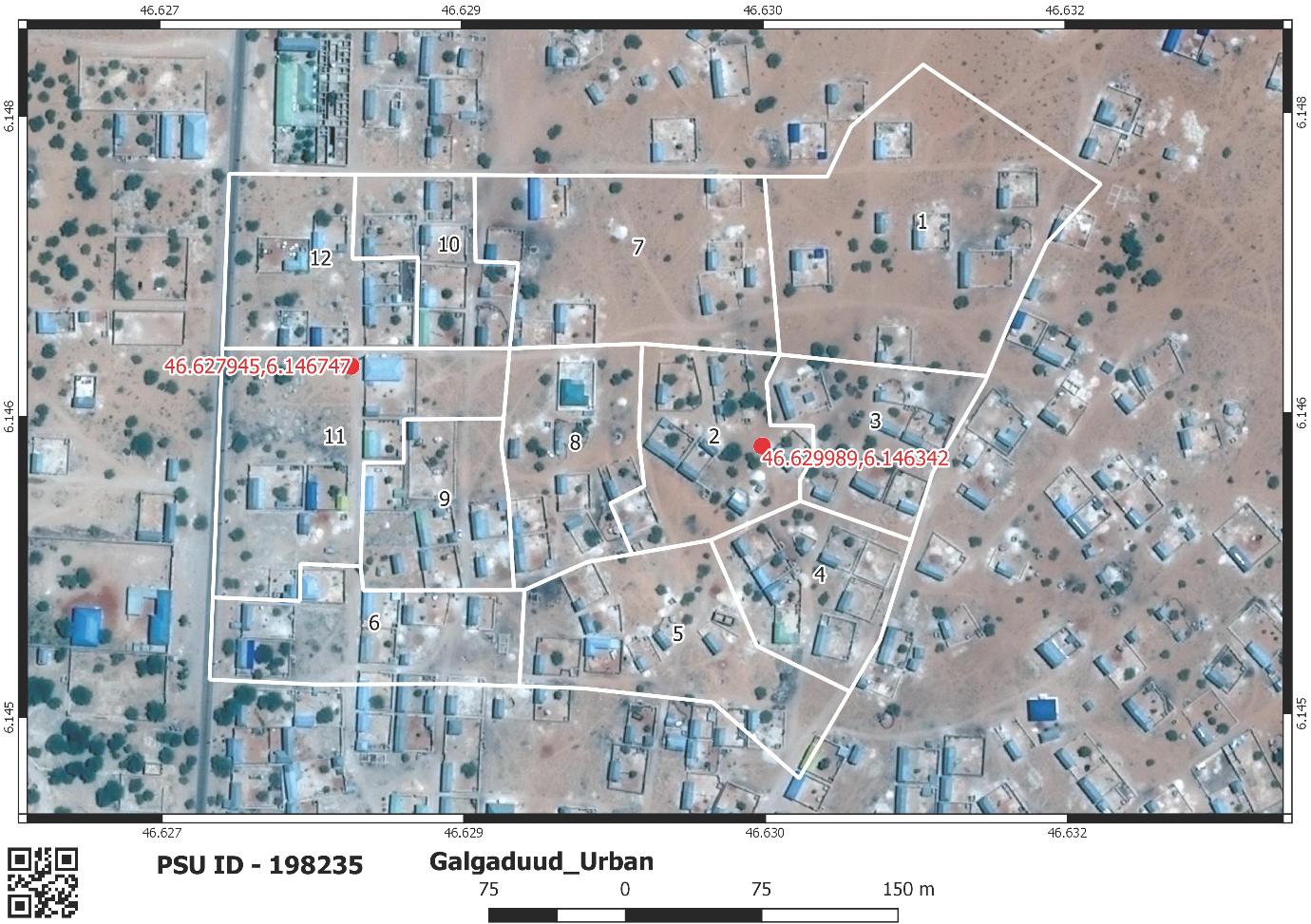


Figure S3. Full map for a PSU ID which has twelve blocks in Galgaduud_urban.


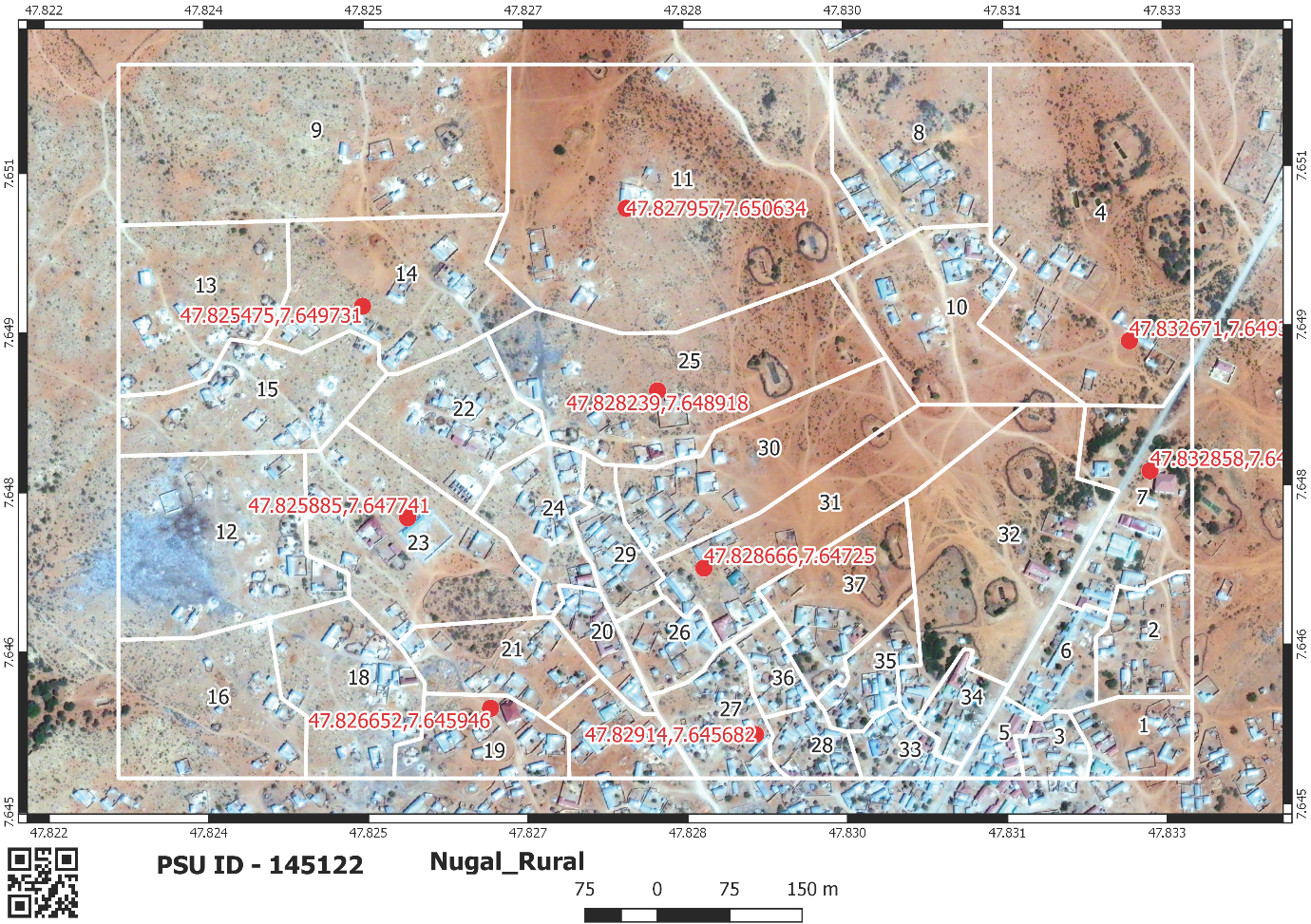


Figure S4. Full map for a PSU which has 37 blocks in Nugal_rural (Over houses)


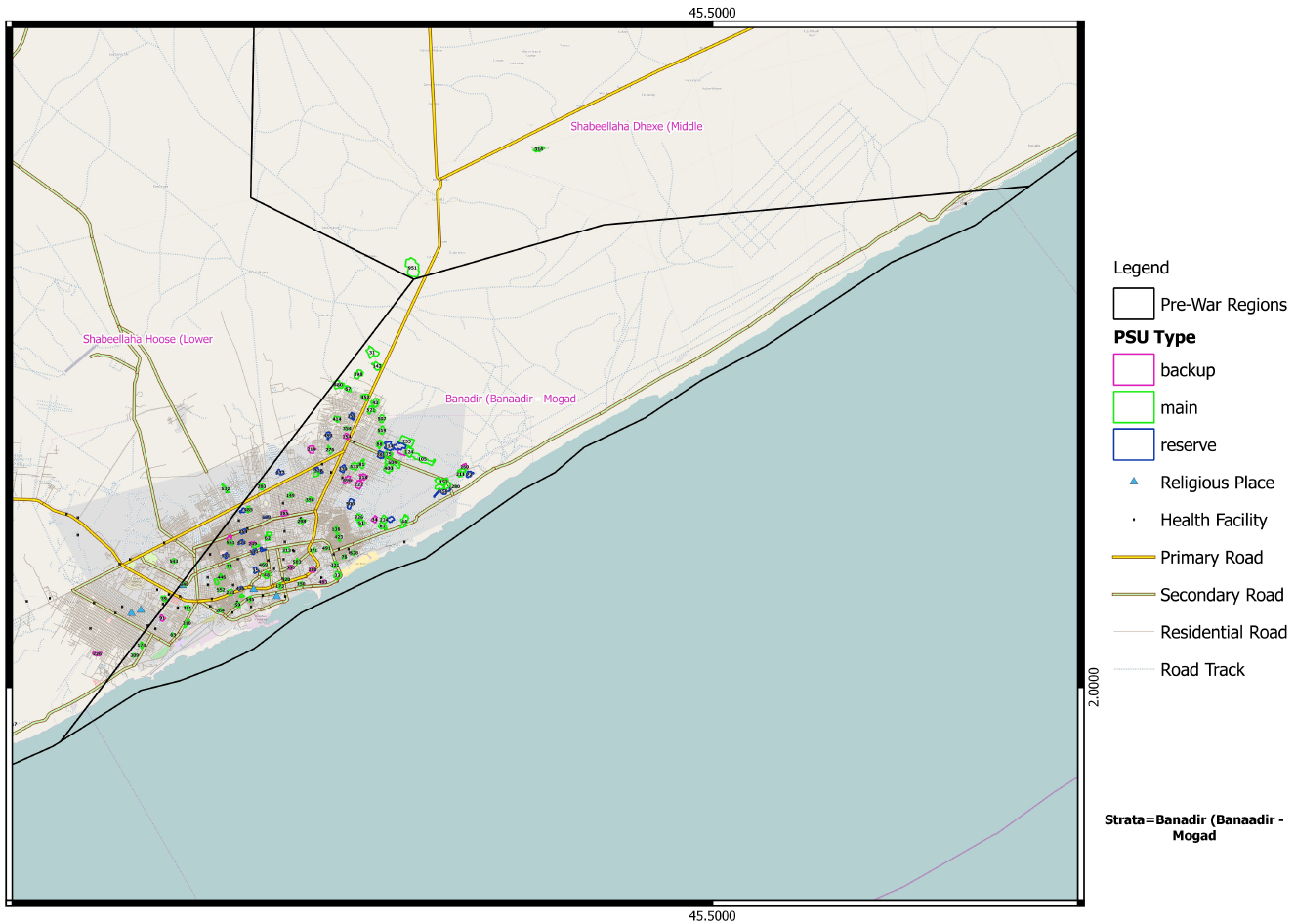


Figure S5. Spatial distribution of PSUs in Mogadishu with necessary georeferenced data for the fieldworkers.

**Reference**

Pape, Utz Johann; Wollburg, Philip Randolph. 2019. Estimation of Poverty in Somalia Using Innovative Methodologies. Policy Research working paper; no. WPS 8735. Washington, D.C. : World Bank Group.

World Bank. 2019. Somali Poverty and Vulnerability Assessment: Findings from Wave 2 of the Somali High Frequency Survey. World Bank, Washington, DC.
